# Effectiveness of a Parent-Focused Intervention Targeting 24-H Movement Behaviors in Preschool-Aged Children: Study Protocol for a Randomized Controlled Trial

**DOI:** 10.3389/fpubh.2022.870281

**Published:** 2022-05-23

**Authors:** Jie Feng, Wendy Yajun Huang, Cindy Hui-Ping Sit

**Affiliations:** ^1^Department of Sport, Physical Education and Health, Hong Kong Baptist University, Kowloon Tong, Hong Kong SAR, China; ^2^Centre for Health and Exercise Science Research, Department of Sport, Physical Education and Health, Hong Kong Baptist University, Kowloon Tong, Hong Kong SAR, China; ^3^Department of Sports Science and Physical Education, The Chinese University of Hong Kong, Shatin, Hong Kong SAR, China

**Keywords:** physical activity, sedentary behavior, sleep, randomized controlled trial, study protocol, preschooler

## Abstract

**Background:**

Interventions targeting single behaviors of preschool-aged children have been mainstream for some time, but integrated interventions targeting all three 24-h movement behaviors (physical activity [PA], sedentary behavior [SB], and sleep hygiene) are less studied. The aims of this study will be to test the feasibility, acceptability, and effectiveness of a parent-focused intervention for preschool-aged children targeting multiple 24-h movement behaviors.

**Methods:**

This three-arm randomized controlled trial will comprise a 12-week intervention and a 12-week follow-up. A total of 150 parent-child dyads will be recruited and randomly allocated to one of three arms: (1) a PA + SB group (dyadic approach), (2) a PA + SB + sleep group (integrated approach), and (3) a wait-list control group. The theory of planned behavior and behavioral change techniques will guide the development of the intervention via workshops, education materials, interactive questionnaires, and reminders. The intervention strategies for the integrated group will be the same as for the dyadic approach except that the intervention will also target sleep hygiene in addition to PA and SB. The primary outcomes will be preschoolers' 24-h movement behaviors (e.g., activity sleep index, compositional data of PA, SB, screen time, and sleep duration). The secondary outcomes will be preschoolers' sleep quality, weight status, cognitive function, and parents' movement behaviors. The feasibility and acceptability of the intervention will also be evaluated.

**Discussion:**

The proposed study will be a theory-based, parent-focused intervention designed to improve all three 24-h movement behaviors among preschoolers. The trial is expected to improve preschoolers' movement behaviors and health outcomes, as well as their parents' movement behaviors. Given the urgent need to promote active lifestyles, our findings will help to determine best practices for movement behavior change among young children.

**Trial Registration:**

The study is prospectively registered at the Chinese Clinical Trial Registry (ChiCTR2200055958).

## Introduction

A healthy lifestyle developed in early childhood is associated with numerous health indicators and tracks into later life (1). Physical activity (PA) (2), sedentary behavior (SB) (3), and sleep (4) in isolation are associated with health in the early years. In addition to their individual effects, the integrated effect of all three movement behaviors in a 24-h day has attracted attention. The World Health Organization (WHO) released 24-h movement guidelines for children under 5 years of age in 2019. Specifically, a healthy day for preschoolers aged between 3 and 4 years should include at least 180 min of PA, of which at least 60 min should be moderate-to-vigorous-intensity PA (MVPA); less than 60 min of sedentary screen time; and 10 to 13 h of good-quality sleep (5). Despite the emerging evidence on 24-h movement behaviors and the promotion of integrated guidelines for the early years, low adherence to the 24-h movement guidelines has been reported in numerous countries (6). In Hong Kong, a recent study found a low compliance rate, with only 2.9% of preschoolers meeting all three 24-h movement guidelines (7). Furthermore, the homeschooling induced by the Coronavirus Disease 2019 (COVID-19) pandemic has led to unfavorable behavior changes. Evidence from several countries indicates that since the outbreak of the pandemic, preschoolers have become more sedentary and had more screen time compared with before the pandemic (8–10). As a result, interventions targeting multiple movement behaviors are warranted and should help to determine best practices for behavior change.

Previous intervention studies have predominantly targeted a single type of movement behavior, particularly PA. Their effectiveness has been summarized in recent systematic reviews (11–13). Interestingly, previous studies have described overflow effects of interventions targeting a single behavior on other non-targeted behaviors. For example, one systematic review reported that interventions aiming to increase PA also reported a decrease in screen time (32 min) for children under 5 years (12). However, given that time is finite and fixed, time spent in PA, SB, and sleep in any 24-h period is not independent and should be viewed as a continuum. In other words, an increase in time spent on one behavior requires an equivalent decrease in time spent on other activities. Studies using statistical models have provided evidence for the effectiveness and flexibility of changing multiple behaviors. Specifically, compositional data analysis found that among adults, similar health outcomes (risk of all-cause mortality) were achieved by different combinations of movement behaviors (14). For example, movement behavior combinations A (light-intensity PA [LPA]: 375 min, MVPA: 3 min, SB: 582 min, sleep: 480 min) and B (LPA: 250 min, MVPA: 55 min, SB: 655 min, sleep: 480 min) during 24 h were associated with a similar risk of all-cause mortality (14). The above evidence suggests that it is necessary to explore whether integrated interventions targeting PA, SB, and sleep simultaneously can generate a larger effect than interventions targeting a single type of behavior. To the best of our knowledge, the only study targeting changes in PA, SB, and sleep simultaneously was conducted in adolescents (15). Compared with the control group, the intervention group had higher PA, lower SB, longer sleep duration, and higher compliance rate to all 24-h movement guidelines after one academic year of intervention (15). However, that study did not evaluate how the 24-h movement behaviors change as a whole, i.e., the composite behavior change. Furthermore, no studies have prospectively targeted changing all 24-h movement behaviors in the early years.

Most behavior change interventions for children under 5 years have been conducted in preschool and childcare settings (11). However, the importance of parental involvement in behavior modifications has been consistently documented (16). A previous systematic review found a greater effect on reducing screen time (−30.6 min/day) for family-based interventions compared to other settings (12). Yoong et al. conducted a study targeting the home routines of preschoolers and found an increase in the sleep of 0.9 h/day after a 3-month intervention (17). Based on the success of family-based interventions targeting single movement behavior, an exploration of the effectiveness of integrated interventions targeting all three 24-h movement behaviors has been recommended (16). Of further note, since the outbreak of the COVID-19 pandemic, preschools have been locked down for a long time and the time that parents spend with their children has significantly increased. A parent-focused intervention to promote 24-h movement behaviors in preschoolers is therefore meaningful and timely.

To fill the research gap and improve understanding of best practices for behavior change in young children, a parent-focused, theory-driven intervention targeting all 24-h movement behaviors for preschool-aged children will be developed. The theory of planned behavior (TPB) will be the theoretical basis for developing this intervention, for several reasons. First, a systematic review found that intention predicted parent-for-child health behaviors, such as reminding and setting limits (18). Second, a study of children in Hong Kong found that parents' perception of the benefits of PA was positively associated with the PA of their children (19). Third, parent-focused TPB-based interventions were effective in improving PA and sleep among preschoolers and children (17, 20). In addition to TPB components, some effective strategies recommended by systematic reviews of interventions in children will be used, such as goal setting, monitoring, and habit development (11, 21, 22). Furthermore, to make the intervention strategies more practical and focused, specific behavior change techniques (BCTs) will be incorporated with the TPB components and strategies (23).

Specifically, the proposed study will (1) test the feasibility and acceptability of a parent-focused intervention targeting multiple 24-h movement behaviors (PA, SB, and sleep hygiene) for preschool-aged children; (2) investigate the effectiveness of a parent-focused intervention to increase PA, reduce SB, and enhance sleep hygiene for preschool-aged children; and (3) examine whether an integrated 24-h movement behavior approach is superior to an intervention targeting daytime behaviors (PA and SB) only. We hypothesize that (1) a parent-focused intervention targeting multiple 24-h movement behaviors for preschool-aged children is feasible and acceptable; (2) both intervention approaches (PA + SB group, PA + SB + sleep group) can effectively change behaviors; and (3) an integrated approach (PA + SB + sleep group) is superior to an intervention targeting daytime behaviors (PA + SB group) only.

## Materials and Methods

### Study Design

**Figure 1:**
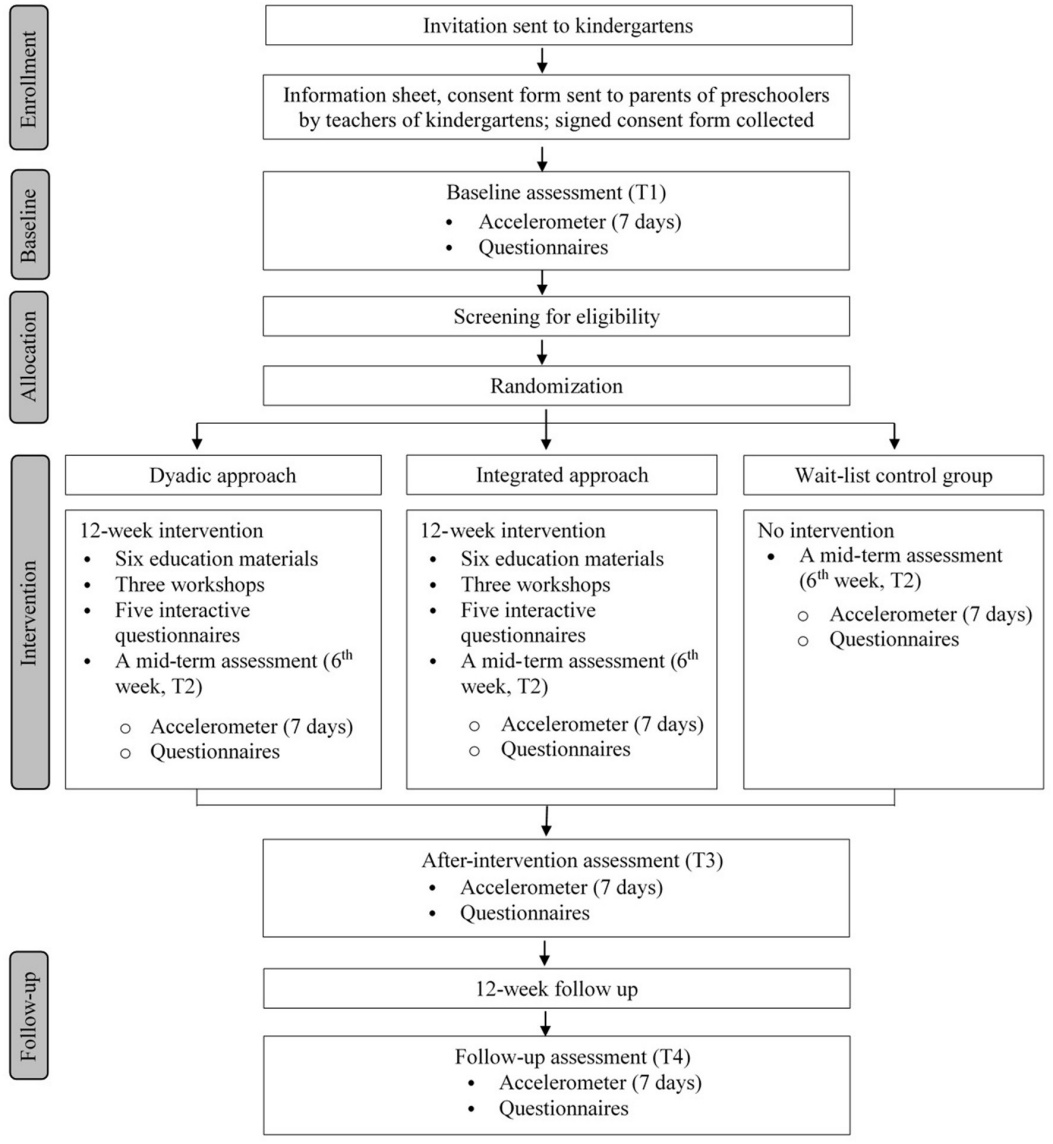
Flow chart of the study.

The study will be a three-arm randomized controlled trial (RCT) using a 1:1:1 allocation ratio, as shown in Figure 1. The intervention will last for 12 weeks and will be followed by a 12-week post-intervention follow-up. After screening and baseline assessments, parent-child dyads will be randomly allocated to one of three groups: (1) PA + SB group (dyadic approach), (2) PA + SB + sleep group (integrated approach), or (3) wait-list control group. Considering the potential impact of the COVID-19 pandemic, the intervention will be delivered predominantly online (Zoom meetings, online survey using Google Forms). The study will follow the Consolidated Standards of Reporting Trials (CONSORT) Statement for randomized trials (http://www.consort-statement.org/).

### Participants

Kindergartens that have participated in our previous studies will be contacted (7), and more families will be invited using purposive and snowball sampling. The specific inclusion criteria are as follows: (1) families having at least one 3-to-5.9-year-old child; (2) the child is living with parents; and (3) the child does not have any diseases that prevent them from participating in PA or falling asleep. Children who meet all of the 24-hour movement guidelines will be excluded. In a previous study conducted in Hong Kong, less than 3% of preschoolers met all three guidelines (7). Therefore, over-recruitment will be necessary to ensure an adequate sample size. For screening, children will wear an ActiGraph accelerometer for seven consecutive days to measure their PA, SB, and sleep, and parents will respond to a question regarding their child's screen time. Written consent will be sought from parents. Approval from the Research Ethics Committee, Hong Kong Baptist University has been obtained (Ref. No.: SOSC-SPEH-2021-22-200).

### Sample Size

A recent systematic review and meta-analysis found a small but statistically significant effect size for interventions to increase PA in children under 5 years old (11). Intervention studies aiming to reduce SB and improve sleep hygiene among young children reported small to medium effect sizes (17, 24). Based on the above information, a prior analysis was conducted, which indicates that a minimum sample size of 99 (33 per group) is required to detect a small effect size *f* of 0.15 with a power of 0.90 under the conditions of an alpha level of 0.05, three arms (PA + SB group, PA + SB + sleep group, wait-list control group), and four repeated measures assuming a correlation between repeated measures at 0.50. Considering a potential attrition rate of 20% (25, 26), 119 children are needed. Therefore, using an over-sampling procedure, 150 parent-child dyads will be recruited and screened. The power calculation is conducted using G^*^Power 3.1.9.7.

### Intervention

#### Procedure

Data collection will be performed during a school visit at baseline (T1), 6 weeks (mid-term; T2), 12 weeks (immediately after the intervention; T3), and 24 weeks (follow-up; T4). Accelerometers and a take-home package will be distributed to parents via the children. The package will include (1) an information sheet about the ActiGraph and instructions on how to wear the device during the 7-day period; (2) online questionnaires to be completed by parents (assessing parents' PA, SB, screen time, and sleep, children's screen time and sleep quality, and demographic information); (3) a log sheet for parents to record when the device is removed and the reasons for removal and their child's nap time. After the baseline and mid-term measurements, a brief report will be provided to parents, including the current movement behaviors of their child and the differences from the recommended durations. After the final measurement (T4), a complete report presenting the changes in the child's movement behaviors will be provided to each parent.

#### PA + SB Group (Dyadic Approach)

Following the baseline assessment and group allocation, families in the PA + SB group will receive educational materials by email. The materials will include information on the WHO recommendations for preschoolers and materials targeting increasing PA, decreasing SB, and screen time for children. The following materials will be delivered to families biweekly to guide parents to set goals for their child: (1) an individual report on the child's current level of movement behaviors (PA, SB, sleep) and divergence from the guidelines; (2) introduction to setting goals and planning (e.g., specific, progressive, achievable); (3) a schedule for parents to fill in, including biweekly goals for each movement behavior; (4) examples to improve PA and decrease screen time (e.g., games designed for children, including written descriptions and pictures); (5) potential barriers and strategies; and (6) strategies for developing habits.

Three online workshops delivered as Zoom meetings will be held for parents in the intervention groups in weeks 1, 3, and 6. The workshops will aim to explain strategies that can be adopted by parents based on BCTs and TPB to solve problems and to encourage parents to comply with the intervention. Specifically, the first workshop will be a brief lecture for parents about the 24-h movement guidelines and the association between movement behaviors and health outcomes, as well as a general introduction to the program (e.g., how to set individual goals, how to use the materials). The second and third workshops will focus on discussing and providing strategies to overcome barriers that the parents have experienced during the intervention, as well as strategies to develop habits. Each workshop will be held as a group meeting of approximately 10 parents to ensure manageability and feasibility. Activities such as check-in polls, topic-based Q & A sessions, and breakout rooms for small-group discussions will be designed to facilitate the workshops. Each workshop will last for 30 to 45 min. If any parents do not have time to attend the workshops, a recorded video of the workshop will be sent to them, and a phone call will be made to provide a brief introduction to the workshop, ask whether they have had any challenges, and suggest strategies. Every 2 weeks, the parents will be asked to complete an online questionnaire using Google Forms. The questions will relate to the children's movement behaviors (e.g., average PA, SB, screen time, bedtime, and wakeup time), whether and how they have used the planning materials for each movement behavior, whether they have achieved the goals and schedules for the coming 2 weeks, etc. At the end of each questionnaire, reminders about the materials use will be presented.

#### PA + SB + Sleep Group (Integrated Approach)

The intervention strategies for the integrated approach group will be the same as for the dyadic approach group except that the intervention content will target sleep hygiene in addition to PA and SB (Table 1). For example, the sleep recommendations for young children and strategies to change parents' practices related to their children's sleep hygiene will be provided.

**Table 1:** Strategies and behavior change techniques involved in the PA + SB + sleep group.

**Approach**	**Time**	**Strategies**	**Behavior change technique (code) (53)**	**Contents**
Education materials	Week 1 & 2	Attitude toward the behavior, subjective norm, monitoring, goal setting	Information about health consequences (5.1) Information of others' approval (6.3) Feedback on behavior (2.2) Discrepancy between current behavior and goal (1.6) Goal-setting (behavior) (1.1)	1. WHO 24-h movement guidelines for preschoolers 2. Benefits of having a healthy lifestyle (i.e., high physical activity, low sedentary behavior, low screen time, sufficient sleep) for preschoolers 3. What important others think about the behavior 4. Individual report on children's current level of movement behaviors and the gap with the guidelines5. Steps and strategies of goal-setting (e.g., SMART goals)
	Week 3 & 4	Planning, intention	Action planning (1.4) Non-specific incentive (10.6)	1. Examples of improving physical activity, reduce sedentary behavior, and improve sleep 2. Examples of giving incentives for child when he/she make effort/progress in performing the behavior
	Week 5 & 6	Perceived behavioral control, planning, intention	Problem-solving (1.2) Action planning (1.4) Non-specific incentive (10.6)	1. Strategies to overcome barriers2. Examples of improving physical activity, reduce sedentary behavior, and improve sleep3. Examples of giving incentives for child when he/she make effort/progress in performing the behavior
	Week 7 & 8	Monitoring, perceived behavioral control, planning, habit development	Feedback on behavior (2.2) Problem-solving (1.2) Action planning (1.4) Habit formation (8.3)	1. Individual report on children's current level of movement behaviors and the gap with the guidelines2. Strategies to overcome barriers 3. Examples of improving physical activity, reduce sedentary behavior, and improve sleep 4. Examples of habits
	Week 9 & 10	Planning, habit development	Action planning (1.4) Behavioral substitution (8.2) Habit formation (8.3)	1. Examples of improving physical activity, reduce sedentary behavior, and improve sleep 2. Strategies for developing habits (substitution, repetition)
	Week 11 & 12	Monitoring, habit development	Feedback on behavior (2.2) Behavioral substitution (8.2) Habit formation (8.3)	1. Individual report on the change of children's movement behaviors during the past 10 weeks (based on parent-reported interactive questionnaires) 2. Strategies for developing habits (substitution, repetition)
Workshops	Week 1	Subjective norm, attitude toward the behavior, goal setting	Information of others' approval (6.3) Information about health consequences (5.1) Goal-setting (behavior) (1.1)	1. Introduction of the program 2. WHO 24-h movement guidelines for preschoolers 3. Benefits of having a healthy lifestyle (i.e., high PA, low SB, low screen time, sufficient sleep) for preschoolers 4. Steps and strategies of goal setting
	Week 3	Perceived behavioral control, intention	Problem-solving (1.2) Verbal persuasion about capability (15.1)	1. Solve problems that families have faced using materials 2. Provide strategies to parental perceived barriers 3. Sharing experiences 4. Encouraging families to comply with the intervention
	Week 6	Perceived behavioral control, intention, habit development	Problem-solving (1.2) Verbal persuasion about capability (15.1) Habit formation (8.3)	1. Provide strategies to parental perceived barriers 2. Sharing experiences 3. Encouraging families to comply with the intervention 4. Example of habits 5. Strategies for developing habits
Interactive questionnaires	Week 2, 4, 6, 8, & 10	Goal setting, monitoring	Review behavior goals (1.5) Self-monitoring of behavior (2.3)	1. Movement behaviors over the past two weeks (duration, whether achieve the goal or not) 2. Bi-weekly goals for each movement behavior 3. Reminders of materials use

#### Wait-List Control Group

Parents in the wait-list control group will not receive the intervention. They will be given access to the educational materials after the intervention and follow-up.

### Outcomes

#### Primary Outcomes

##### Activity Sleep Index

Similar to the activity-sleep index created for adults (27), a composite movement behavior score will be created to summarize the multiple dimensions of PA, SB, and sleep among preschoolers. The composite movement behavior score will comprise scores for the following six dimensions representing broad variables that have been examined in relation to the 24-h movement guidelines (28, 29) and in previous studies on sleep among young children (30):

1. PA: total time on PA of all intensities.
2. PA: total time on MVPA.
3. Screen time.
4. Sleep: bedtime duration (time in bed).
5. Sleep: sleep onset variability (variability in sleep onset times).
6. Sleep: morning wake-time variability (variability in wake times).

Each item will be rescaled from 0 to 10, with a higher score indicating healthier movement behaviors. The formula for rescaling is as follows (27):


$$\begin{array}{l} Rescaled\;score\;=\;\left(\frac{\left(X-X_{\min}\right)}{X_{Range}}\right)\times 10 \end{array}$$

where *X* is the observed value, *X*_min_ is the minimum value observed, and *X*_range_ is the difference between the maximum and minimum values observed (27). A total score ranging from 0 to 60 will be obtained by summing the scores for all items, with higher scores indicating healthier movement behaviors.

##### Compositional Data

Compositional data analysis has been suggested to examine movement behaviors occurring within a finite period, specifically when they are continuously measured using accelerometers (31). The time spent on one movement behavior relative to all other behaviors will be presented as isometric log-ratio coordinates (*ilr*), which will be the outcome variable in the regression models. For example, a 24-h period can be divided into time spent in PA, SB, and sleep. Taking PA as an example, the equation is as follows:


$$\begin{array}{l} ilr_{1}=\sqrt{\frac{2}{3}}\ln\left(\frac{PA}{\sqrt[{2}]{SB^{*}Sleep}}\right) \end{array}$$

where *ilr*_1_ represents sleep relative to all other movement behaviors.

The time allocated to SB relative to other movement behaviors can be assessed using the *ilr*_2_, which can be expressed as follows:


$$\begin{array}{l} ilr_{2}=\sqrt{\frac{2}{3}}\ln\left(\frac{SB}{\sqrt[{2}]{PA^{*}Sleep}}\right) \end{array}$$

Similarly, the time allocated to sleep relative to other movement behaviors can be assessed using *ilr*_3_, which can be expressed as follows:


$$\begin{array}{l} ilr_{3}=\sqrt{\frac{2}{3}}\ln\left(\frac{Sleep}{\sqrt[{2}]{PA^{*}SB}}\right) \end{array}$$

##### Children's Physical Activity and Sedentary Behavior

ActiGraph accelerometers (ActiGraph, Pensacola, Florida, USA) will be used to assess children's PA and sedentary time. During a school visit, trained researchers will help children attach the ActiGraph to their non-dominant wrist using a wrist band (32). Children will be asked to wear the device for seven consecutive days during 24-h period. The ActiGraph should be removed when children undertake any water-based activities (e.g., bathing and swimming), and parents will be asked to complete a log sheet to record the time and reasons for removing the device. The ActiGraph data will be collected at a sampling rate of 30 Hz (32). Data will be downloaded using ActiLife software v6.13.4. and analyzed in 5-s epochs. A time period of more than 60 min of zero counts continuously will be regarded as non-wear time. Days with at least 16 hours recorded as wearing time will be regarded as valid (33). To reflect children's habitual behavior patterns, only children who provide data for at least 3 days including at least 1 weekend day will be included in the final analysis (34). Cut-off points that have been validated in preschool-aged children will be used. Specifically, sedentary time, LPA, and MVPA will be defined as ≤ 328 counts, 329–1392 counts, and ≥1393 counts per 5 seconds for vector magnitude, respectively (32). Total PA (TPA) will be calculated by summing LPA and MVPA.

##### Children's Screen Time

Screen time will be reported by parents using a question modified from the Children's Leisure Activities Study Survey questionnaire–Chinese version (CLASS-C) (35): How much time does your child spend on sedentary screen time (e.g., watching TV/DVDs, playing video games, using computers, using tablets and mobile phones)?

##### Children's Sleep Duration

Sleep duration will be assessed using ActiGraph data matched with a parent-reported diary and nap schedule provided by kindergartens. The duration of nighttime sleep will be estimated using ActiLife software v6.13.4 in 60-s epochs. The automated Sadeh et al. algorithm (36) will be applied to identify bedtime and wakeup time, and the Tudor-Locke algorithm (37) will be used to detect periods of sleep. For the duration of daytime naps, bedtime and wakeup time will be identified using ActiLife software and matched with the nap schedule provided by kindergartens and log sheets completed by parents. The total sleep duration in a day will be defined as the sum of nighttime sleep and daytime naps.

#### Secondary Outcomes

##### Children's Sleep Quality

Children's sleep quality will be measured using the Chinese version of the Children's Sleep Habits Questionnaire (CSHQ) (38), which has been used to measure Chinese preschoolers' sleep patterns and problems based on parental reports. The CSHQ consists of 33 items in eight dimensions: bedtime resistance, sleep onset delay, sleep duration, sleep anxiety, night waking, parasomnias, sleep-disordered breathing, and daytime sleepiness. Three options are provided for each item: 5 to 7 times a week (scored 3), 2 to 4 times a week (scored 2), and 0 to 1 time a week (scored 1). Higher scores indicate greater sleep problems. The Chinese version of the CSHQ has shown good reliability (Cronbach's α = 0.73) and validity (38).

##### Children's Body Mass Index

Preschoolers' heights and weights will be measured by trained researchers during school visits. Children's body mass index (BMI) will be calculated as weight (kg)/height (m^2^).

##### Parents' Physical Activity and Sedentary Behavior

The Chinese version of the International Physical Activity Questionnaire–Short Form (IPAQ-SF) (39) will be used to estimate parents' PA (walking, moderate-intensity PA [MPA], vigorous-intensity PA [VPA], total energy expenditure) and SB over the previous seven days. Energy expenditure corresponding to walking, MPA, and VPA are 3.3 metabolic equivalents (METs), 4.0 METs, and 8.0 METs, respectively. The sum of energy expenditure on walking, MPA, and VPA will be considered total energy expenditure (MET-minutes/week).

##### Parents' Screen Time

Parents will respond to two questions: (1) on average, in a 24-h day, how much time do you usually spend on sedentary screen time (e.g., using computers, tablets, and mobile phones) for work, and (2) how much time do you usually spend on sedentary screen time (e.g., watching TV/DVDs, playing video games, using computers, using tablets, and mobile phones) for leisure and entertainment?

##### Parents' Sleep Duration and Quality

Parents' sleep duration and quality over the previous month will be measured using the Chinese version of the Pittsburgh Sleep Quality Index (PSQI) (40). The PSQI consists of 18 self-reported items in seven dimensions: sleep quality, sleep latency, sleep duration, habitual sleep efficiency, sleep disturbance, sleep medication used, and daytime dysfunction. Four options scored from 0 to 4 are provided for each item and a higher PSQI score indicates worse sleep quality. The PSQI has shown good reliability (Cronbach's α = 0.84) and validity among Chinese adults (40).

##### Cognitive Function

The Childhood Executive Functioning Inventory (CHEXI) (41) will be used to evaluate the executive function of the preschoolers. The CHEXI consists of 24 items in two domains: working memory (13 items) and inhibition (11 items). For each item, five options ranging from “1 = definitely not true” to “5 = definitely true” are provided, where a higher score indicates worse executive function. The CHEXI has demonstrated high reliability and has been translated into traditional Chinese (42). It can be downloaded freely online (https://chexi.se/onewebmedia/CHEXI-TraditionalChinese2016.pdf).

##### Covariates

Preschoolers' characteristics (age, sex, number of siblings), parents' characteristics (age, sex, height, weight, education level), family income, family size, family structure, and type of residence will be reported by parents. Children's eating habits will be measured using questions based on a previous study conducted among preschoolers in Hong Kong (43). Five domains (fruit, vegetables, dairy products, breakfast, high-energy-density foods) will be covered using five items. Responses to all items will be made on a 5-point Likert scale. For example, breakfast intake will be assessed using one question: How often does your child eat breakfast? Possible answers will be as follows: <1 day/week, 1–2 days/week, 3–4 days/week, 5–6 days/week, and every day. Possible answers for the other questions will be <1 serving/day, 1–2 servings/day, 3–4 servings/day, 5–6 servings/day, and >6 servings/day.

##### Feasibility and Acceptability

**Feasibility**. Retention rates for each group and reasons given by participants who remove, withdraw, or are lost to follow-up will be recorded. Adverse effects, if any, will be documented.

**Fidelity and Adherence**. Intervention fidelity will be monitored, including the delivery of three workshops, six educational materials, two brief reports of measurements, and five questionnaires and reminders (one brief questionnaire every 2 weeks) for each person (24).

**Acceptability**. Based on a previous study (17), five items will be used to separately measure the PA and SB components for the two intervention groups. For the integrated approach, an additional five items will be used to evaluate the sleep component. Among the five items for each component, one item will be used to evaluate the overall intervention (poor, average, or good). The other four items will be used to evaluate each strategy separately (i.e., educational material, workshop, report of measurements, interactive questionnaire and reminder about material use). Parents will be asked to rate the usefulness using 5-point Likert scales (from “1 = not useful” to “5 = very useful”).

### Randomization and Blinding

Randomization and group assignment will be conducted using computer-generated random numbers by a third person who is blinded to the purpose of the study. After baseline data collection, the participants will be randomly allocated to one of three arms. The trained staff who will be responsible for data collection will also be blinded to group assignment. Although blinding participants to the intervention is not feasible for this study, families in the two intervention groups will not be disclosed of the differences in the intervention contents.

### Statistical Methods

Analyses will be performed using SPSS 27.0. The significance level will be set at 0.05. Descriptive statistics will be presented for the children's baseline movement behaviors. Feasibility and acceptability will be presented using percentages. Differences in movement behaviors across three groups at baseline will be assessed using analysis of covariance. The intention-to-treat principle (all participants) and sensitivity analysis (participants with complete data) will be used to compare primary outcomes between groups. Generalized estimating equations will be applied to examine the effects of the intervention (PA + SB group vs. control group, PA + SB + sleep group vs. control group) on primary outcomes (children's overall movement behaviors, total PA, MVPA, sedentary time, screen time, sleep duration) and secondary outcomes, adjusting for covariates. Also, the difference of the effect between two intervention groups (PA + SB group vs. PA + SB + sleep) will be examined using generalized estimating equations.

## Discussion

The aim of the proposed study will be to explore the impact of an integrated movement behaviors (PA + SB + sleep) intervention on promoting overall 24-h movement behaviors (based on activity sleep index and compositional data) and to evaluate how changes in overall 24-h movement behaviors mediate the effect of interventions on health outcomes among preschoolers. At the time this study was initiated, to the best of our knowledge, no studies have examined the effect of an intervention targeting change in all 24-h movement behaviors among preschoolers.

Engaging in a healthy combination of movement behaviors has been related to health among young children (1). In Hong Kong, low compliance with the WHO 24-h movement guidelines among preschoolers has been reported, suggesting the need for interventions aimed at improving all three behaviors (7). Although previous interventions have been demonstrated to be effective in improving individual behaviors among preschoolers (11–13), no study has simultaneously targeted all three 24-h movement behaviors using integrated strategies for the early years. To fill this gap, the proposed intervention will target all three behaviors, and we will examine how these behaviors change as a whole. Two indicators—an activity sleep index and compositional behavior data—will be used to examine preschoolers' overall behaviors. The activity sleep index provides a total score for different dimensions of movement behaviors, with a higher score indicating better overall movement behaviors (27). This indicator may therefore better reflect the overall pattern of 24-h movement behaviors (total PA, MVPA, screen time, and duration and timing of sleep) and can provide insight into how different behaviors change simultaneously. The other primary outcome will be compositional data that reveal the time spent on one behavior relative to other behaviors, which has been increasingly used (31). Previous studies have used compositional data analysis when examining 24-h movement behaviors and health outcomes among preschool-aged children, but most of them applied cross-sectional designs and measured sleep duration subjectively (44–47). Therefore, an intervention study adopting device-based measurement is needed to objectively explore the effect on compositional behavior data in this age group.

In addition to improving children's movement behaviors, our secondary outcomes will be the changes of children's health-related outcomes after the intervention. We will focus on two health indicators: body weight status and cognitive function. Obesity has become a global health issue, and 39 million 0-to-5-year-olds were categorized as overweight or obese in 2020 (48). Therefore, not surprisingly, body weight status has been the most commonly examined indicator in previous studies examining the associations between meeting the 24-h movement guidelines and health outcomes in the early years (6). Cognitive function, which is supported by brain development and linked to future academic performance, develops rapidly during the preschool period (49). Systematic reviews have reported that PA interventions benefit children's cognitive functions consistently (2, 50), but studies of SB and sleep interventions that examine cognitive function in children are limited (3, 4). Despite the importance of healthy body weight and cognitive function for preschoolers, there has been no convincing evidence of the relationship between adherence to movement guidelines and these two indicators because of the limited number of studies and the cross-sectional designs used in most of them (6). In addition, a recent systematic review called for experimental studies to explore the effects on health of meeting the guidelines (6). The proposed study will respond to this demand. It is expected that children in both intervention groups will show better health and children in the integrated intervention group will have superior improvement.

Through this intervention, we will also aim to help parents develop a healthier lifestyle. Evidence from a systematic review suggests that there are small but positive associations between parents' and children's movement behaviors, based on a majority of cross-sectional studies (51). A consensus statement on the importance of the family to the movement behaviors of children and youth noted that parents can support their children by improving their lifestyles in various ways, including modeling and co-participation (16). An ongoing 6-month-intervention that aims to increase children's PA will evaluate parents' PA, but no results are available yet (52). Thus, there is a need to explore the changes in parents' movement behaviors after our proposed interventions. We hypothesize that parents in the intervention groups will improve their movement behaviors.

The proposed study has numerous strengths, including targeting all three 24-h movement behaviors, the application of a theoretical framework, objective measurement of children's movement behaviors, and an RCT design. The proposed intervention is parent-focused and will explore an integrated approach targeting all three movement behaviors for preschool-aged children. The findings will help to determine best practices for behavior change. The beneficiaries will include families with young children and other stakeholders in early education.

## Ethics Statement

The studies involving human participants were reviewed and approved by Research Ethics Committee, Hong Kong Baptist University.

## Author Contributions

WH and JF conceptualized and designed the study. CS contributed to concept and design refinement. JF prepared the first draft under the supervision of WH. All authors contributed to revising the manuscript and approving the final manuscript.

## Conflict of Interest

The authors declare that the research was conducted in the absence of any commercial or financial relationships that could be construed as a potential conflict of interest.

## Publisher's Note

All claims expressed in this article are solely those of the authors and do not necessarily represent those of their affiliated organizations, or those of the publisher, the editors and the reviewers. Any product that may be evaluated in this article, or claim that may be made by its manufacturer, is not guaranteed or endorsed by the publisher.
